# Topology of syngameons

**DOI:** 10.1002/ece3.3507

**Published:** 2017-11-01

**Authors:** William J. Boecklen

**Affiliations:** ^1^ Department of Biology New Mexico State University Las Cruces NM USA

**Keywords:** hybridization propensity, hybridization rate, interspecific hybridization, syngameon

## Abstract

Syngameons are sets of species linked by interspecific hybridization. Common observations regarding the structure of syngameons are that hybridization propensity is not uniform across species and that patterns of hybridization are dominated by a few species. I use computer simulations to test these claims in naturally occurring syngameons selected from the literature and from personal observation. Natural syngameons, especially those involving plants, typically exhibit nonrandom structure: The first three order statistics for the number of hybrid partners and the variance in the number of hybrid partners are larger than chance alone would predict. The structure of two insect syngameons examined is not significantly different from random. To test a hypothesis that variation in hybridization propensity across species in natural syngameons is simply an artifact of hybridization opportunity, I examine the structure of four artificial syngameons (fertility relationships) produced by full diallel crosses. Three of four artificial syngameons exhibit nonrandom structure, as the observed variation in number of successful crosses is larger than chance alone would predict. In general, there are no significant results involving the order statistics. Finally, I discuss biogeographic, ecological, and phylogenetic hypotheses for variation in hybridization propensity across species in natural syngameons.

## 1 INTRODUCTION

Interspecific hybridization is a common feature in eukaryotic evolution and it has important consequences to cladogenesis, species interactions, invasion dynamics, and conservation biology. Recent evidence suggests that it has played a role in human evolution, as modern humans hybridized with both Denisovans and Neanderthals (Sankararaman, Mallick, Patterson, & Reich, 2016). In addition, interspecific hybridization has played a central role in the development of evolutionary theory, as early interest in interspecific hybrids (reviewed by Focke (1913) and Marza & Cerchez (1967)) contributed to the development of species concepts (Linnaeus, 1744; Lotsy, 1925), genetics (Mendel & Bateson, 1925; Naudin, 1862), plant reproductive biology (Gärtner, 1849; Roberts, 1919), and speciation (Linnaeus, 1760). More recently, introgressive hybridization has been examined with respect to adaptive radiation (Seehausen, 2004) and invasion dynamics (Blair, Blumenthal, & Hufbauer, 2012; Ellstrand & Schierenbeck, 2000). In addition, plant hybrid zones have become important model systems to examine tritrophic interactions involving plants, herbivorous insects, and parasitoids (Aguilar & Boecklen, 1992; Preszler & Boecklen, 1994). There continues to be much interest among evolutionary biologists in interspecific hybridization, particularly with respect to speciation in animals (Dowling & Secor, 1997; Seehausen, 2004; Willis, van Oppen, Miller, Vollmer, & Ayre, 2006; Zinenko, Sovic, Joger, & Gibbs, 2016), fungi (Giraud, Refrégier, Le Gac, de Vienne, & Hood, 2008; Restrepo, Tabima, Mideros, Grünwald, & Matute, 2014), and plants (Grant, 1981; Rieseberg, 1997; Soltis & Soltis, 2009). There has been an exponential increase in the number of publications on interspecific hybridization over the last 30 years (Schwenk, Brede, & Streit, 2008), and the topic has been the subject of many recent reviews (Abbott et al., 2013; Mallet, 2005; Seehausen, 2004; Soltis & Soltis, 2009; Whitney, Ahern, Campbell, Albert, & King, 2010; Willis et al., 2006).

Patterns of interspecific hybridization exhibit several emergent properties. For animals, the probability of hybridization between species may be inversely related to their phylogenetic distances (Coyne & Orr, 1989; Tubaro & Lijtmaer, 2002). For plants, the probability of homoploid versus polyploid hybrid speciation appears to be positively associated with the extent of genetic divergence of hybridizing species (Buggs, Soltis, & Soltis, 2011; Chapman & Burke, 2007; Paun, Forest, Fay, & Chase, 2009). In addition, the occurrence of hybrids varies by taxonomic group, with approximately 25% of plant species and 10% animal species producing natural hybrids (Mallet, 2005; Rieseberg, 1997). Lastly, there may be a strong phylogenetic signal to hybridization propensity, as natural hybrids are not equally distributed among families and genera of vascular plants (Ellstrand, Whitkus, & Rieseberg, 1996; Whitney et al., 2010).

**Figure 1:**
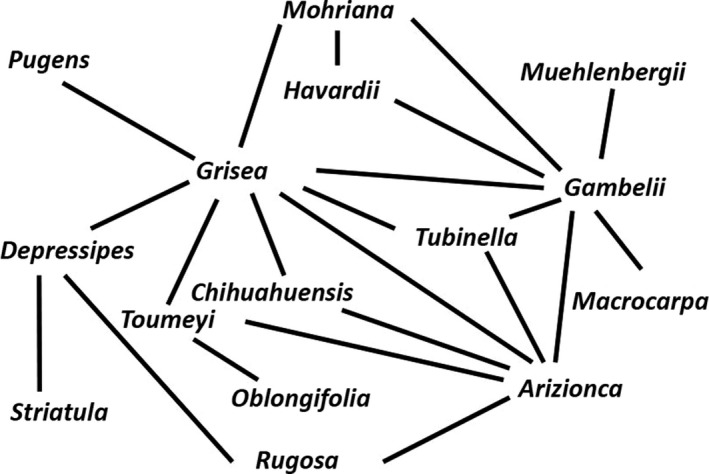
Pattern of hybridization between white oaks of the Southwestern United States and Northern Mexico

A syngameon is produced when a group of closely related species forms a complex set of hybrid combinations (Lotsy, 1925). Classic examples include irises of the California Pacific Coast (Lenz, 1959), white oaks of the Eastern United States (Hardin, 1975), and British species of *Potamogeton* (Clapham, Tutin, & Warburg, 1962). Syngameons also exhibit emergent properties; a common observation is that hybridization events are not equally distributed among species and that a few species dominate the pattern of hybridization. Consider the pattern of hybridization between southwestern white oaks as depicted by a network graph (Figure 1) – the set of hybridizations is dominated by three species: *Quercus gambelli*,* Q*. *arizonica*, and *Q*. *grisea*; and the number of hybrid partners ranges from 8 (*Q*. *grisea*) to 1 (*Q*. *striatula*).

Variation between species within a syngameon in hybridization propensity may be due to a number of factors related to biogeography, ecology, phylogeny, reproductive biology, and genetics. Alternatively, the pattern may be due to chance alone. The appearance of a structured pattern of hybridization within a given syngameon may simply be an artifact of the number of species involved and the number of hybrid combinations. Nonrandom structure in the pattern of interspecific hybridization among closely related species addresses a fundamental question in interspecific hybridization, namely “Why do some species readily hybridize, while others do not.” The first step in answering this question is to demonstrate that there are, in fact, nonrandom patterns of hybridization. To date, this has not been done.

To reject chance in favor of more biologically interesting mechanisms producing patterns of hybridization within syngameons, it is necessary to enumerate all possible network graphs constrained by the observed number of species and hybrid combinations. The observed structure of a given syngameon can then be compared to the sample space of possible network graphs. I use computer simulations to determine whether syngameons, as represented by network graphs, exhibit nonrandom structure. In particular, I examine order statistics and variation in the number of hybridization partners to determine whether hybridizations events are significantly concentrated in a few species and whether the variation in hybrid propensity is greater than chance alone would predict. The objective was to place observations regarding the structure of syngameons on a sound probabilistic foundation.

## 2 MATERIALS AND METHODS

I examined eight naturally occurring syngameons (Table 1). Two of these involve insects (carabid beetles and heliconiine butterflies) (Kubota & Sota, 1998; Mallet, Beltrán, Neukirchen, & Linares, 2007); the rest involve plants. Four of the plant syngameons were selected from the literature (Clayberg, 1968; den Nijs & Visser, 1985; Raamsdonk, Wietsma, & Vries, 1992; Sorensson & Brewbaker, 1994); two are unpublished (Southwestern White Oaks and *Boechera*). I have extensive experience with the Southwestern White Oak syngameon, having examined plant‐herbivore interactions (Gaylord, Preszler, & Boecklen, 1996; Yarnes & Boecklen, 2005), reproductive biology (Williams, Boecklen, & Howard, 2001), and plant photochemistry (Yarnes, Boecklen, & Salminen, 2007; Yarnes, Boecklen, Tuominen, & Salminen, 2006). The *Boechera* syngameon represents an ongoing collaboration to examine hybridization propensity as a function of genetic distance (D. Bailey, pers. com.). For the *Boechera* syngameon, I examined a set of species that could potentially hybridize and a subset of species that actually do hybridize (*Boechera* subset). For the Heliconius Butterflies syngameon, I considered naturally occurring hybrids alone and then supplemented with artificial hybrids (Mallet et al., 2007).

**Table 1:** Natural and artificial syngameons examined for nonrandom structure based on order statistics and standard deviations of observed numbers of hybrid combinations

Taxa	Species	Hybrid combinations	Hybridization rate	Order statistics			*SD*	Hybrids	Reference
				1	2	3			
				Observed	Observed	Observed	Observed		
				Expected	Expected	Expected	Expected		
				*p*‐value	*p*‐value	*p*‐value	*p*‐value		
Eastern White Oaks	16	38	0.317	11	11	8	3.357	Natural	Hardin (1975)
				7.9	7.0	6.5	1.721		
				.012	<.001	.024	<.001		
Southwestern White Oaks	15	22	0.210	8	7	6	2.282	Natural	R. Spellenberg *(pers. com*.)
				5.6	4.8	4.3	1.451		
				.022	.004	.006	.001		
*Boechera*	58	42	0.025	8	7	5	1.789	Natural	Alexander et al. (2015)
				4.7	4.0	3.7	1.172		D. Bailey (*pers. com*.)
				.002	<.001	<.001	<.001		
Boechera (subset)	35	42	0.071	8	7	5	1.735	Natural	Alexander et al. (2015)
				5.9	5.1	4.7	1.462		D. Bailey (*pers. com*.)
				.045	.014	.616	.059		
*Asplenium*	16	19	0.158	7	6	4	2.062	Natural	Brownsey (1977)
				5.0	4.2	3.7	1.347		
				.039	.009	.710	.002		
*Potamogeton*	19	18	0.105	6	5	4	1.792	Natural	Clapham et al. (1962)
				4.5	3.7	3.3	1.253		Grant (1981)
				.093	.063	.274	.006		
California Irises	12	14	0.211	5	4	3	1.371	Natural	Lenz (1959)
				4.5	3.8	3.3	1.277		Grant (1981)
				.469	.736	.998	.397		Young (1998)
Carabid Beetles	16	9	0.075	3	3	2	0.957	Natural	Kubota and Sota (1998)
				3.1	2.4	2.1	0.974		
				.846	.409	.995	.616		
Heliconius Butterflies	9	9	0.250	5	3	2	1.323	Natural	Mallet et al. (2007)
				3.8	3.1	2.6	1.127		
				.111	.954	1.000	.277		
Heliconius Butterflies	11	13	0.236	6	4	3	1.567	Natural + Artificial	Mallet et al. (2007)
				4.5	3.7	3.2	1.258		
				.082	.685	.995	.142		
*Sinningia*	15	32	0.305	8	8	6	2.282	Artificial	Clayberg (1968)
*Rechsteineria*				7.3	6.4	5.9	1.651		
				.349	.035	.778	.020		
*Leucaena*	12	47	0.712	11	10	10	1.946	Artificial	Sorensson and Brewbaker (1994)
				10.0	9.4	9.0	1.418		
				.191	.390	.081	.034		
*Allium*	7	16	0.761	6	6	5	1.272	Artificial	Van Raamsdonk et al. (1992)
				5.8	5.2	5.0	0.946		
				.777	.248	.987	.146		
*Cucumis*	11	23	0.418	8	6	6	2.183	Artificial	den Nijs and Visser (1985)
				6.6	5.8	5.2	1.456		
				.106	.691	.249	.008		

To test a hypothesis that structure in natural syngameons is simply a function of variation among species in the opportunity to hybridize, I conducted two separate analyses. First, I examined four artificial syngameons (fertility diagrams) selected from the literature and produced by full diallel crosses. Because each species was mated to all other species, hybridization opportunity was equal for all species. The structure of these artificial syngameons was analyzed as described below. Second, I examined the relationship between geographic range and hybridization propensity in the *Boechera* syngameon. I assumed, all other things being equal, that geographic range is related to the opportunity to hybridize. I examined county‐level species occurrences for *Boechera* species throughout the Southwestern United States (Alexander et al., 2015). I used nonparametric regression to determine whether the number of hybrid partners was related to the number of counties occupied by a species.

I recorded from each natural and artificial syngameon the total number of species, the total number of hybrid combinations, and the number of hybrid combinations for each species. I calculated the hybridization rate for a syngameon as the number of hybrid combinations observed relative to the number possible. I then calculated the order statistics for each syngameon. The first‐order statistic is the number of hybrid combinations exhibited by the species with the most hybrid combinations, the second‐order statistic is the number of hybrid combinations exhibited by the species with the next most hybrid combinations, and so on. For example, the first‐order statistic for the Southwestern White Oak syngameon is 8, while the second‐order statistic is 7 (Figure 1). To examine aggregate patterns of hybridization within syngameons, I calculated the standard deviation in the number of hybrid partners across all species in the syngameon.

For each syngameon, I used computer simulations to randomly assign the observed total number hybridization events between the observed total numbers of species. I then calculated all order statistics and the standard deviation in hybrid partners. I repeated each simulation 5,000 times and developed a sampling distribution for each order statistic and for the standard deviation in hybrid partners. I used the sampling distributions to determine the statistical significance of the observed order statistics and standard deviations.

## 3 RESULTS

There was considerable variation in the structure of natural syngameons (Table 1). Numbers of species participating in a syngameon ranged from 9 (California Irises syngameon) to 58 (*Boechera* syngameon), while hybridization rates ranged from 0.024 (*Boechera* syngameon) to 0.317 (Eastern White Oak syngameon). Syngameons involving plants typically were more complex than were those involving insects; plant syngameons generally contained more species and more hybrid combinations.

**Figure 2:**
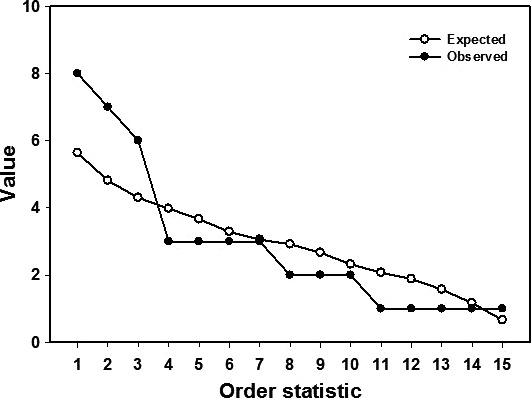
Observed and expected order statistics for the number of hybrid combinations between species in the Southwestern White Oak syngameon

Computer simulations indicated that significant nonrandom structure was a general feature of the natural syngameons, at least for those involving plants. The results are exemplified by the Southwestern White Oak syngameon (Figure 1). The first several order statistics for the number of hybrid combinations by species were significantly larger than were those expected by chance alone (Figure 2). This pattern held for all but one of the natural plant syngameons (California Iris syngameon). Observed values of the order statistics and observed values of the standard deviations in number of hybrid partners were significantly larger than chance alone would predict (Table 1). (The first two order statistics for the *Potamogeton* syngameon were weakly significant, while the standard deviation in number of hybrid partners was highly significant.) The two insect syngameons did not exhibit nonrandom structure; none of the order statistics or standard deviations in the number of hybrid partners were significantly different from that expected by chance alone. Adding artificial hybrids to the Heliconius Butterflies, syngameon did not produce significant nonrandom structure.

As expected, the four artificial syngameons exhibited higher hybridization rates than did the natural syngameons. However, in no case were all possible hybrid combinations realized. Hybridization rates for the artificial syngameons averaged 0.549 and ranged from 0.305 (*Sinningia* and *Rechsteineria* syngameon) to 0.761 (*Allium* syngameon). A Mann–Whitney U‐test indicated a highly significant difference between artificial and natural syngameons in hybridization rates (*p* = .009).

Computer simulations indicated significant nonrandom structure in three of the four artificial syngameons. While the order statistics generally were not significantly different from that expected by chance alone, the standard deviations in the number of hybrid partners were. Only the *Allium* syngameon did not exhibit nonrandom structure (Table 1).

Nonparametric regression indicated a marginally significant relationship (*p* = .052) between the number of hybrid partners and number of counties occupied for species in the *Boechera* syngameon. Geographic distribution explained roughly 26% of the variation in hybridization propensity.

## 4 DISCUSSION

I have demonstrated that artificial and natural syngameons, at least for those involving plants, exhibit nonrandom structure with respect to variation in hybridization propensity. I also have demonstrated that patterns of hybridization within these syngameons are typically dominated by a few species. I found no evidence that syngameons involving animals, in this case insects, exhibited patterns of hybridization that differed from those that chance alone would predict. Of course, the sample size for animal syngameons was quite small, and an adequate determination of nonrandom structure in animal syngameons must await a larger compendium of case studies.

Unresolved is the mechanism that produces nonrandom structure in syngameons. For natural syngameons, differences in the geographic distributions of species are a compelling candidate mechanism. Geographically widespread species simply have more opportunities for hybridization than do geographically restricted species. I found partial support for this hypothesis in the *Boechera* syngameon, as there was a marginally significant positive relationship between a species’ geographic range and number of hybrid‐producing mating combinations. I recognize that the measure of geographic range, the number of counties occupied, is not optimal. A rigorous test regarding the relationship between geographic range and hybridization propensity must await a more thorough analysis.

Analysis of the artificial syngameons suggests that patterns of hybridization within syngameons are not wholly a function of mating opportunity. The fertility diagrams that I analyzed were produced by full diellel crosses – every species had equal opportunity to hybridize. Nevertheless, I detected significant variation between species in hybridization propensities. This suggests that there are factors intrinsic to species that make them greater or lesser hybridizers. Exactly what these factors are is an open question. Possible factors include plasticity in habitat requirements, timing, and duration of reproductive episodes, ploidy levels (plants), strength of species recognition, and intricacies of mating displays (animals), specificity of sperm‐egg interactions, and cytoplasmic incompatibility (animals). This list is not exhaustive, nor is it mutually exclusive. Of course, the opportunity hypothesis and the intrinsic‐factors hypothesis are not mutually exclusive either.

One potential intrinsic factor that deserves greater investigation is phylogenetic position. It is known at a gross level that there is a phylogenetic signature to hybridization propensities. Plants produce natural hybrids more readily than do animals (Mallet, 2005), and production of natural hybrids is not equally distributed across orders of vascular plants (Ellstrand et al., 1996; Whitney et al., 2010). At a finer level, the probability of interspecific hybridization may be related to genetic distance (Chapman & Burke, 2007; Coyne & Orr, 1989). Avid hybridizers may be those species with many close neighbors in genetic space, or they may typically occupy basal or distal positions within their respective clades. A robust test of this hypothesis will require a large compendium of case studies with detailed phylogenies that include all members of a syngameon.

## CONFLICT OF INTEREST

None declared.

## AUTHOR CONTRIBUTION

WJB analyzed and interpreted all literature and unpublished data, wrote the computer code for the simulations, and drafted and revised the article.

## References

[ece33507-bib-0001] Abbott, R. , Albach, D. , Ansell, S. , Arntzen, J. W. , Baird, S. J. E. , Bierne, N. , … Zinner, D. (2013). Hybridization and speciation. Journal of Evolutionary Biology, 26, 229–246. 23323997 10.1111/j.1420-9101.2012.02599.x

[ece33507-bib-0002] Aguilar, J. M. , & Boecklen, W. J. (1992). Patterns of herbivory in the *Quercus grisea* × *Quercus gambelii* species complex. Oikos, 64, 498–504.

[ece33507-bib-0003] Alexander, P. J. , Windham, M. D. , Beck, J. B. , Al‐Shehbaz, I. A. , Allphin, L. , & Bailey, C. D. (2015). Weaving a tangled web: Divergent and reticulate speciation in *Boechera fendleri* Sensu Lato (Brassicaceae: Boechereae). Systematic Botany, 40, 572–596.

[ece33507-bib-0004] Blair, A. C. , Blumenthal, D. , & Hufbauer, R. A. (2012). Hybridization and invasion: An experimental test with diffuse knapweed (*Centaurea diffusa* Lam.). Evolutionary Applications, 5, 17–28. 25568026 10.1111/j.1752-4571.2011.00203.x PMC3353329

[ece33507-bib-0501] Brownsey, P. J. (1977). A taxonomic revision of New Zealand species of *Asplenium* . New Zealand Journal of Botany, 15, 39–86.

[ece33507-bib-0005] Buggs, R. J. A. , Soltis, P. S. , & Soltis, D. E. (2011). Biosystematic relationships and the formation of polyploids. Taxon, 60, 324–332.

[ece33507-bib-0006] Chapman, M. A. , & Burke, J. M. (2007). Genetic divergence and hybrid speciation. Evolution, 61, 1773–1780. 17598755 10.1111/j.1558-5646.2007.00134.x

[ece33507-bib-0007] Clapham, A. , Tutin, T. G. , & Wargurg, E. E. (1962). Flora of the British Isles, 2nd ed Cambridge: Cambridge University P.

[ece33507-bib-0008] Clayberg, C. D. (1968). Biosystematic studies in *Sinningia* and *Rechsteineria* (Gesneriaceae). American Journal of Botany, 55, 829–833.

[ece33507-bib-0009] Coyne, J. A. , & Orr, H. A. (1989). Patterns of speciation in Drosophila. Evolution, 43, 362–381. 28568554 10.1111/j.1558-5646.1989.tb04233.x

[ece33507-bib-0010] den Nijs, A. P. M. , & Visser, D. L. (1985). Relationships between African species of the genus *Cucumis* L. Estimated by the production, vigour and fertility of F1 hybrids. Euphytica, 34, 279–290.

[ece33507-bib-0011] Dowling, T. E. , & Secor, C. L. (1997). The role of hybridization and introgression in the diversification of animals. Annual Review of Ecology and Systematics, 28, 593–619.

[ece33507-bib-0012] Ellstrand, N. C. , & Schierenbeck, K. A. (2000). Hybridization as a stimulus for the evolution of invasiveness in plants? Proceedings of the National Academy of Sciences of the United States of America, 97, 7043–7050. 10860969 10.1073/pnas.97.13.7043 PMC34382

[ece33507-bib-0013] Ellstrand, N. C. , Whitkus, R. , & Rieseberg, L. H. (1996). Distribution of spontaneous plant hybrids. Proceedings of the National Academy of Sciences of the United States of America, 93, 5090–5093. 11607681 10.1073/pnas.93.10.5090 PMC39411

[ece33507-bib-0014] Focke, W. O. (1913). History of plant hybrids. The Monist, 23, 396–416.

[ece33507-bib-0015] Gärtner, C. F. (1849). Versuche und Beobachtungen über die Bastarderzeugung im Pflanzenreich. Stuttgart: Georuckt Bei, K. F. Herring & Co.

[ece33507-bib-0016] Gaylord, E. S. , Preszler, R. W. , & Boecklen, W. J. (1996). Interactions between host plants, endophytic fungi, and a phytophagous insect in an oak (*Quercus grisea* x *Q. gambelii*) hybrid zone. Oecologia, 105, 336–342. 28307106 10.1007/BF00328736

[ece33507-bib-0017] Giraud, T. , Refrégier, G. , Le Gac, M. , de Vienne, D. M. , & Hood, M. E. (2008). Speciation in fungi. Fungal Genetics and Biology, 45, 791–802. 18346919 10.1016/j.fgb.2008.02.001

[ece33507-bib-0018] Grant, V. (1981). Plant speciation, 2nd ed New York, NY, USA: Columbia University Press.

[ece33507-bib-0019] Hardin, J. W. (1975). Hybridization and introgression in *Quercus alba* . Journal of the Arnold Arboretum, 56, 336–363.

[ece33507-bib-0020] Kubota, K. , & Sota, T. (1998). Hybridization and speciation in the carabid beetles of the subgenus Ohomopterus. Researches on Population Ecology, 40, 213–222.

[ece33507-bib-0021] Lenz, L. W. (1959). Hybridization and speciation in the pacific coast Irises. Aliso 4, 237–309.

[ece33507-bib-0022] Linnaeus, C. (1744). De Peloria. Upsaliae, Upsaliae.

[ece33507-bib-0023] Linnaeus, C. (1760). Disquisitio de sexu plantarum. Academia Imperiali Scientiarum Petropolitana praemio ornata. Amoenitates Academicae 10, 100–131.

[ece33507-bib-0024] Lotsy, J. P. (1925). Species or linneon? Genetica, 7, 487–506.

[ece33507-bib-0025] Mallet, J. (2005). Hybridization as an invasion of the genome. Trends in Ecology & Evolution, 20, 229–237. 16701374 10.1016/j.tree.2005.02.010

[ece33507-bib-0026] Mallet, J. , Beltrán, M. , Neukirchen, W. , & Linares, M. (2007). Natural hybridization in heliconiine butterflies: The species boundary as a continuum. BMC Evolutionary Biology, 7, 28. 17319954 10.1186/1471-2148-7-28 PMC1821009

[ece33507-bib-0027] Marza, V. D. , & Cerchez, N. (1967). Charles Naudin, a pionneer of contemporary biology. Journal d'agriculture Traditionnelle et de Botanique Appliquée, 14, 369–401.

[ece33507-bib-0028] Mendel, G. , & Bateson, W. (1925). Experiments in plant‐hybridisation. Cambridge, MA: Harvard University Press.

[ece33507-bib-0029] Naudin, C. (1862). Nouvelles recherches sur l'hybridité dans les végétaux. Annales des sciences naturelles. Botanique, ser 4, 19, 180–203.

[ece33507-bib-0030] Paun, O. , Forest, F. , Fay, M. F. , & Chase, M. W. (2009). Hybrid speciation in angiosperms: Parental divergence drives ploidy. New Phytologist, 182, 507–518. 19220761 10.1111/j.1469-8137.2009.02767.x PMC2988484

[ece33507-bib-0031] Preszler, R. W. , & Boecklen, W. J. (1994). A three‐trophic‐level analysis of the effects of plant hybridization on a leaf‐mining moth. Oecologia, 100, 66–73. 28307028 10.1007/BF00317131

[ece33507-bib-0032] Raamsdonk, L. W. D. V. , Wietsma, W. A. , & Vries, J. N. D. (1992). Crossing experiments in *Allium* L. section Cepa. Botanical Journal of the Linnean Society, 109, 293–303.

[ece33507-bib-0033] Restrepo, S. , Tabima, J. F. , Mideros, M. F. , Grünwald, N. J. , & Matute, D. R. (2014). Speciation in fungal and Oomycete plant pathogens. Annual Review of Phytopathology, 52, 289–316. 10.1146/annurev-phyto-102313-050056 24906125

[ece33507-bib-0034] Rieseberg, L. H. (1997). Hybrid origins of plant species. Annual Review of Ecology and Systematics, 28, 359–389.

[ece33507-bib-0035] Roberts, H. F. (1919). The contribution of Carl Friedrich von Gartner to the history of plant hybridization. American Naturalist, 53, 431–445.

[ece33507-bib-0036] Sankararaman, S. , Mallick, S. , Patterson, N. , & Reich, D. (2016). The combined landscape of Denisovan and Neanderthal ancestry in present‐day humans. Current Biology, 26, 1241–1247. 27032491 10.1016/j.cub.2016.03.037 PMC4864120

[ece33507-bib-0037] Schwenk, K. , Brede, N. , & Streit, B. (2008). Introduction. Extent, processes and evolutionary impact of interspecific hybridization in animals. Philosophical Transactions of the Royal Society of London. Series B, Biological Sciences, 363, 2805–2811. 18534946 10.1098/rstb.2008.0055 PMC2453525

[ece33507-bib-0038] Seehausen, O. (2004). Hybridization and adaptive radiation. Trends in Ecology & Evolution, 19, 198–207. 16701254 10.1016/j.tree.2004.01.003

[ece33507-bib-0039] Soltis, P. S. , & Soltis, D. E. (2009). The role of hybridization in plant speciation. Annual Review of Plant Biology, 60, 561–588. 10.1146/annurev.arplant.043008.092039 19575590

[ece33507-bib-0040] Sorensson, C. T. , & Brewbaker, J. L. (1994). Interspecific compatibility among 15 *Leucaena* species (Leguminosae: Mimosoideae) via artificial hybridizations. American Journal of Botany, 81, 240–247.

[ece33507-bib-0041] Tubaro, P. L. , & Lijtmaer, D. A. (2002). Hybridization patterns and the evolution of reproductive isolation in ducks. Biological Journal of the Linnean Society, 77, 193–200.

[ece33507-bib-0042] Whitney, K. D. , Ahern, J. R. , Campbell, L. G. , Albert, L. P. , & King, M. S. (2010). Patterns of hybridization in plants. Perspectives in Plant Ecology, Evolution and Systematics, 12, 175–182.

[ece33507-bib-0043] Williams, J. H. , Boecklen, W. J. , & Howard, D. J. (2001). Reproductive processes in two oak (Quercus) contact zones with different levels of hybridization. Heredity, 87, 680–690. 11903563 10.1046/j.1365-2540.2001.00968.x

[ece33507-bib-0044] Willis, B. L. , van Oppen, M. J. H. , Miller, D. J. , Vollmer, S. V. , & Ayre, D. J. (2006). The role of hybridization in the evolution of reef corals. Annual Review of Ecology Evolution and Systematics, 37, 489–517.

[ece33507-bib-0045] Yarnes, C. T. , & Boecklen, W. J. (2005). Abiotic factors promote plant heterogeneity and influence herbivore performance and mortality in Gambel's oak (*Quercus gambelii*). Entomologia Experimentalis et Applicata, 114, 87–95.

[ece33507-bib-0046] Yarnes, C. T. , Boecklen, W. J. , & Salminen, J.‐P. (2007). No simple sum: Seasonal variation in tannin phenotypes and leaf‐miners in hybrid oaks. Chemoecology, 18, 39–51.

[ece33507-bib-0047] Yarnes, C. T. , Boecklen, W. J. , Tuominen, K. , & Salminen, J.‐P. (2006). Defining phytochemical phenotypes: Size and shape analysis of phenolic compounds in oaks (Fagaceae, *Quercus*) of the Chihuahuan Desert. Canadian Journal of Botany, 84, 1233–1248.

[ece33507-bib-0502] Young, N. D. (1998). Pacific Coast Iris delineation using three species definitions: biological, phylogenetic and genealogical. Biological Journal of the Linnean Society, 63, 99–120. 9480733

[ece33507-bib-0048] Zinenko, O. , Sovic, M. , Joger, U. , & Gibbs, H. L. (2016). Hybrid origin of European Vipers (*Vipera magnifica* and *Vipera orlovi*) from the Caucasus determined using genomic scale DNA markers. BMC Evolutionary Biology, 16, 76. 27068498 10.1186/s12862-016-0647-7 PMC4828770

